# Evidence supporting the use of a subjective staff evaluation to assess the benefit of rehabilitation in hemodialysis patients undergoing inpatient rehabilitation

**DOI:** 10.1186/s12882-020-02118-8

**Published:** 2020-11-09

**Authors:** Takahiro Shimoda, Helen Alston, Angelique Chai, Sarbjit V. Jassal

**Affiliations:** 1 Department of Rehabilitation Sciences, Kitasato University Graduate School of Medical Sciences, Sagamihara, Japan; 2 Division of Nephrology, University Health Network, Toronto, Canada

**Keywords:** ADL, Hemodialysis patients, QOL, Rehabilitation, Staff subjective evaluation

## Abstract

**Background:**

We questioned whether the introduction of a subjective evaluation of patient-specific goals, could be used as a valid method to assess the effectiveness of inpatient rehabilitation.

**Methods:**

In this prospective cohort study, all admissions to the UHN hemodialysis rehabilitation service between April 2013 and August 2016 were included. We introduced a system of subjective assessment, performed by the team at the time of admission and discharge. We evaluated Functional Independence Measure (FIM®) score and KDQoL for objective measures of physical function and patient-reported quality of life.

**Results:**

A total of 201 patients were included. The median FIM score at discharge correlated well with the subjective staff evaluation. FIM score changes for those with evaluations for *Success*, *Partial success,* and *Not Successful* were 28 [interquartile range (IQR) 20–34], 24 [IQR18–31], 16 [IQR 11–34] respectively. The median PCS at discharge for those deemed to have *Success was* 37.4 [IQR31.0, 44.7], and for those with *Partial success* & *Not Successful* 28.8 [IQR 22.4, 39.2]. There was no correlation with MCS scores (55.2 [IQR 51.2, 60.2], 58.4 [IQR 50.1, 63.1] respectively).

**Conclusions:**

These results suggest the subjective staff evaluation is a brief but valid assessment of patient outcome for dialysis patients undergoing inpatient rehabilitation.

## Background

The goal of rehabilitation is to optimize an individual’s functioning despite disease, injury or other health conditions. Specific activity goals are determined in the context of the individual’s lifestyle and resources, as well as with regard to how they interact with their physical, attitudinal and social environment [1]. Thus when evaluating whether a period of rehabilitation has gone well, it is important to include the individual expectations for their own lifestyle together with their ability to complete functional tasks. To capture patient-specific goals into the evaluation of rehabilitation outcomes, we established a process where goals were identified and reassessed, subjectively, by the clinicians at both admission and discharge. This subjective-staff evaluation was applied to all admissions in a unit providing inpatient rehabilitation care to patients maintained chronically on hemodialysis (HD).

The objective of this study was to evaluate if the subjective staff evaluation was validated by objective measures of rehabilitation success (determined from the Functional Independence Measure (FIM®) score [2]) and by changes in patient-reported quality of life scores (measured using the KDQoL [3]).

## Methods

Data were obtained from an ongoing prospective cohort study of patients undergoing rehabilitation and hemodialysis (HD) at the Toronto Rehabilitation Institute, University Health Network, Toronto. All admissions to the hemodialysis rehabilitation service between April 2013 and August 2016 were included. Patient characteristics were determined from the prospectively-maintained clinical database and included age, sex, time on renal replacement therapy, cause of renal failure, reason for rehabilitation, comorbidity condition, hemoglobin, albumin, serum creatinine, and depressive symptoms. The study was approved by the University Health Network research ethics board. All patients received a thorough explanation of the study and only patients able and willing to provide written consent were included.

At the time of admission, realistic goals were set by the rehabilitation clinicians and the patient collectively, and documented in the chart (Appendix). At the time of discharge, each goal was evaluated, and compared to the patient’s function at that time. The team collectively assessed the proportion of patient-specific rehabilitation goals that had been met into three groups: < 30% (subjective evaluation ‘*not successful*’); 30–69% (subjective evaluation ‘*partial succes*s’); ≥70% (subjective evaluation ‘*success’*). To ensure team consensus, a minimum of three different health care disciplines had to be involved in the discharge evaluation (occupational therapy/ physiotherapy/ recreational therapy/ rehabilitation nurse/ unit physicians).

The objective change in physical function, attributable to rehabilitation, was quantified using the change in FIM® [2] score over the admission period. Admission and discharge FIM® scores are collected routinely in the minimal dataset for centers providing active inpatient rehabilitation in Ontario. The score is a validated measure of independence in core daily activity, and comprises 13 motor items and 5 cognitive items, with each item worth 7 points (1 - complete assistance, to 7 - complete independence). A maximal score of 126 is consistent with independence in all activities of daily living, while 18 is the lowest score possible.

A subset of patients also completed the KDQoL-36 [3] at both admission and discharge. The KDQoL-36 has 36 items that include all items within the ShortForm-12 (SF-12) generic quality of life scale, plus 24 disease-specific kidney-related items. KDQoL results are often further subdivided into physical component scores (PCS) and mental component scores (MCS).

Demographic and clinical data were reported using mean and standard deviation for parametric data or median and interquartile range (IQR) if non-parametric. Categorical variables were reported as a percentage. Differences were assessed using unpaired T-tests (for parametric variables), or Mann–Whitney U test (non parametric). Categorical data were assessed using the chi-squared test. The association between subjective staff evaluation scores and the change in physical function measured by change in FIM®; and the change in QOL score was assessed using the Kruskal-Wallis test. All analyses were performed using the R statistical software (version 3.3.0; R Foundation for Statistical Computing, Vienna, Austria). In all analyses, *P* < 0.05 was taken to indicate statistical significance.

## Results

**Fig. 1:**
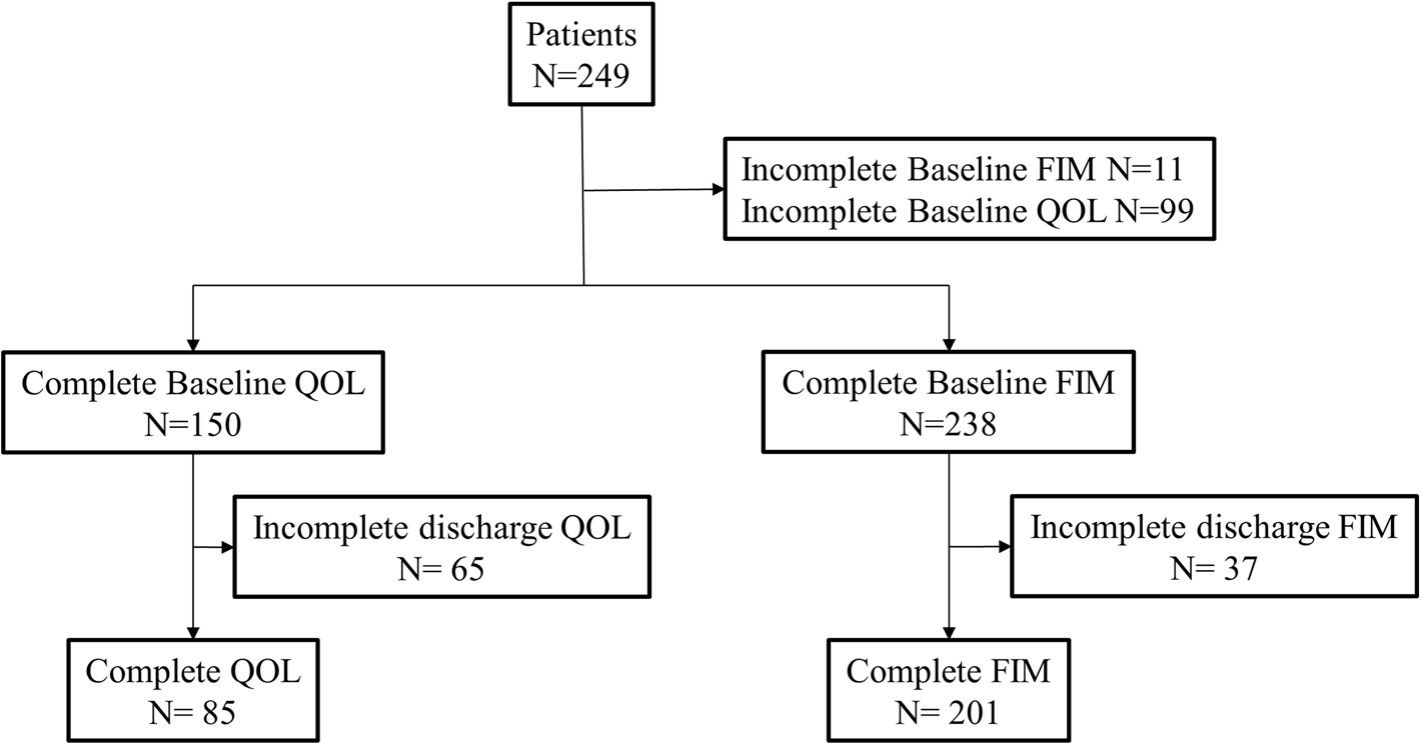
Flow diagram showing Study recruitment

A total of 249 patients underwent rehabilitation and dialysis over the 35 month study period. Of these 11 had an incomplete FIM at baseline and 37 had an incomplete FIM at discharge (Fig. 1) resulting in a total of 201 participants with complete FIM results. Patients who had a complete follow up FIM® were mostly male (57%, *n* = 115), with a mean age of 71.5 years (interquartile range, 64.9–78.9) (Table 1). One hundred forty- three of the patients underwent rehabilitation for acute hospitalization, 59 were admitted for rehabilitation after an acute fall. The median length of stay was 41 days (IQR 31–47). More than 80% of patients succeeded in ≥70% of the goals identified at admission (*n* = 163, 81.1%). Of the remaining patients 30 (14.9%) and 8 (4.0%), achieved 30–69% of goals or < 30% of goals respectively. The median FIM® score at the time of admission was 77 (*n* = 238, IQR 68–89) and at discharge 110 (*n* = 201, IQR 97–117)). Ninety-nine patients did not give consent to participation in the quality of life assessment. As only one patient with complete KDQOL data fell in the *Not Successful* group, data for those with Partial Success and Not Successful were condensed into one group for all analyses involving KDQOL data.

**Table 1:** Demographic and clinical details of study population

	Target Study population	Complete FIM data	Complete KDQoL data
	*N* = 249	*N* = 201	*N* = 85
Age, year	71.7 [64.9–79.1]	71.5 [64.9–78.9]	68.9 [63.9–76.7]
Female, %	102 (41.0)	86 (42.8)	36 (42.4)
Dialysis vintage, %			
< 3 months	78 (31.3)	68 (33.8)	30 (35.3)
3 to12 months	31 (12.4)	21 (10.4)	12 (14.1)
12 < months	140 (56.2)	112 (55.7)	43 (50.6)
Cause of Renal Failure, %			
DM and HT/vascular	126 (50.6)	106 (52.7)	38 (44.7)
GN	31 (12.4)	25 (12.4)	14 (16.5)
Other	81 (32.5)	59 (29.4)	25 (29.4)
Modified Charlson score, score	6 [3–8]	6 [4–8]	5 [3–7] ^a^
Length of stay	41 [31–47]	42 [33–47]	41 [31–46]
Medical Reason for Rehabilitation, %			
Acute hospitalization	143 (57.4)	111 (55.2)	51 (60.0)
Fall	59 (23.7)	51 (25.4)	18 (21.2)
Other	47 (18.9)	39 (19.4)	16 (18.8)
Albumin, g/L	31 [28–34]	31 [29–34]	31 [28–34]
Creatinine, mg/L	478 [350–602]	491 [358–611]	503 [370–611]
Hemoglobin, g/L	96 [87–105]	96 [87–103]	95 [87–102]

Values are shown as median [IQR] or n (%)

a: *p* < 0.05 vs Target Study population

**Fig. 2:**
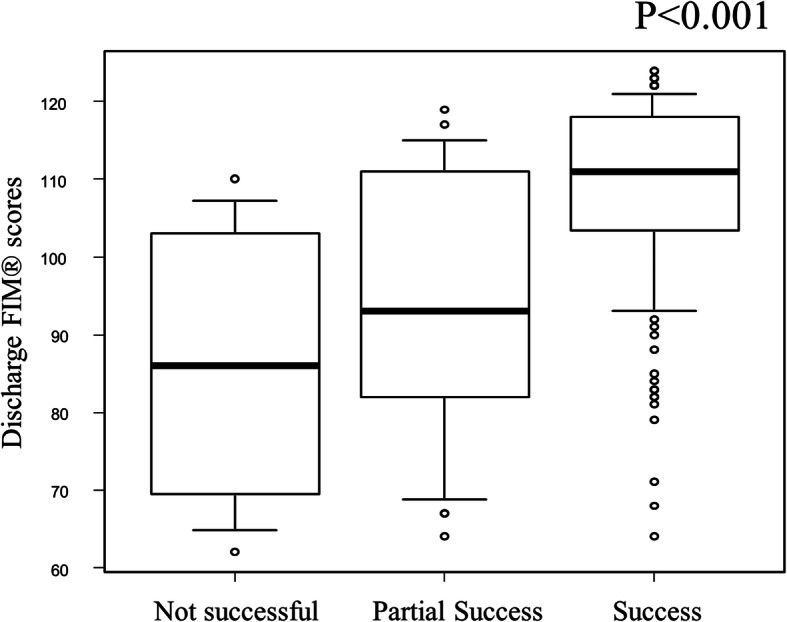
Box plot showing the relationship between discharge FIM® scores and the subjective staff evaluation

The subjective staff evaluation corresponded well with discharge FIM® scores (*Success*: 111 [IQR 104–118], *Partial Success*: 93 [IQR 82–109], *Not Successful*: 86 [71–102], Fig. 2; *P* < 0.001). At the time of discharge, patients with a subjective staff evaluation “success” had higher quality of life scores than those in the other groups although this was largely due to changes in physical component scores (median PCS scores of 37.4 [IQR 31.0–44.7] compared to 28.8 [IQR 22.4–39.2] for those in the combined *Partial Success & Not Successful* group). MCS scores at discharge were similar across groups 55.2 [IQR 51.2–60.2] and 58.4 [IQR 50.1–63.1] respectively.

**Fig. 3:**
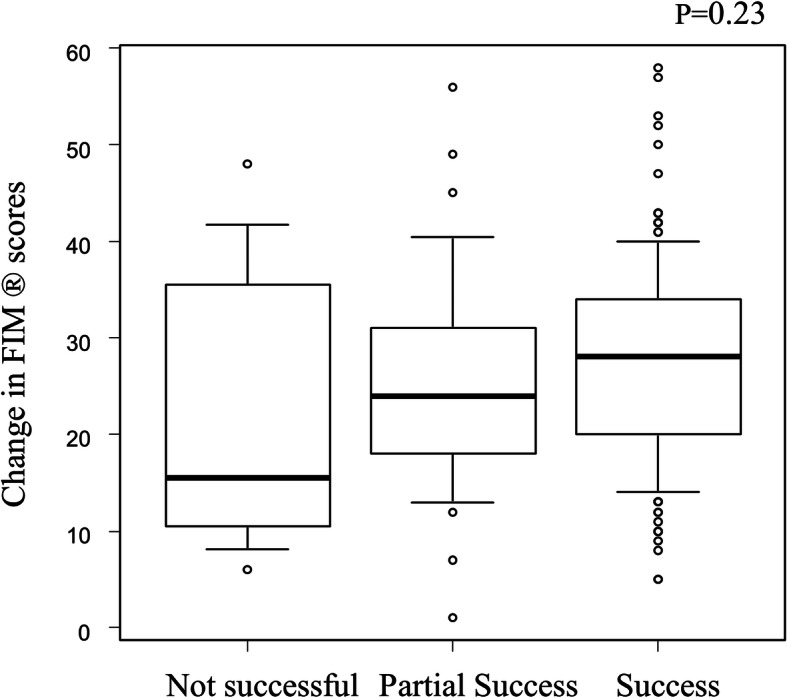
Box plot showing the relationship between the change in FIM® score and subjective staff evaluation

**Fig. 4:**
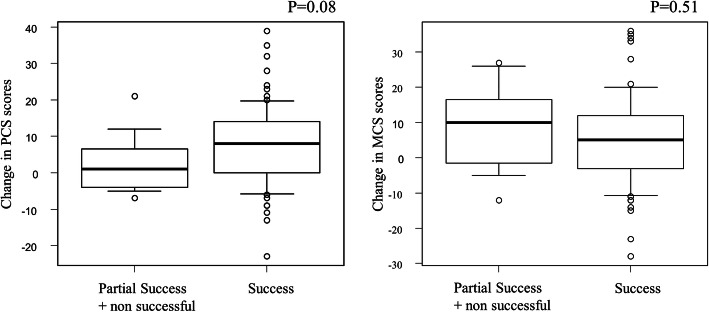
Box plot showing the change in both the Physical Component Score (PCS) and Mental Component Scores (MCS) measured on the KDQOL-36 and subjective staff evaluation

The subjective staff evaluation correlated only weakly with absolute change in FIM® scores (*Success*: 28 [IQR 20–34], *Partial Success*: 24 [IQR 18–31], *Not Successful*: 16 [IQR 11–34], Fig. 3) and in PCS scores (*Success*: 8 [IQR 0–14], *Partial Success & Not Successful*: 1 [IQR -4 – 7]) and MCS scores (*Success*: 10 [IQR -2 – 17], *Partial Success & Not Successful*: 5 [IQR -3 – 12] (Fig. 4). However when defined using a threshold for improvement, more patients reported substantial improvements in quality of life (defined as > 5 point change in PCS scores over time) in those with *Success* than the combined *Partial Success & Not Successful* group (for PCS change *n* = 44, 60% vs. n = 4, 40% respectively). A similar trend was not seen with change in baseline MCS change (*n* = 38, 51% vs *n* = 6, 60% respectively).

## Discussion

The data presented in this study suggest that the routine use of a staff subjective evaluation is a valid quick assessment tool that corresponds with objective measures of physical function and patient-reported quality of life. The assessment is rapid and easily applied and has the added advantage of being specific to an individual’s needs and goals. We therefore suggest it may be an appropriate method to assess the success of our rehabilitation program although acknowledge that further work is required to assess if it can be used to estimate prognosis and long term outcomes. Similar methods have been proposed in other areas of rehabilitation such as paediatric rehabilitation, neurorehabilitation and in those using prosthetics for example [4, 5]. However in a geriatric dialysis population, the use of traditional goal attainment scaling methods may be more challenging due to the multiple dimensions of goals, and the challenge of concomitant medical instability.

We chose to study a small group of hemodialysis patients undergoing rehabilitation as part of a project to assess the value of subjective team evaluation. Although dialysis patients have a unique and often complex pattern of comorbidity, the nature of their impairment and disability is similar to the general rehabilitation population. Consistent with this expectation, recent studies show the magnitude of improvement with rehabilitation, in patients recently started onto dialysis, to be similar to that of non-dialysis patients [6–8]. The study population has similar characteristics to other studies of dialysis patients suggesting low risk of selection bias and that our findings may be widely generalizable [9–21]. For example, Forrest et al., reported that the median change in FIM scores, in 40 HD patients, with a mean age of 69 years, undergoing inpatient rehabilitation was similar to our population (a change from mean baseline scores of 80 to 104 by discharge) [11]. Our multidisciplinary team is highly experienced at obtaining detailed information about the patient’s characteristics, physical function, social factors and environmental factors, and therefore at setting realistic rehab goals for each patient. As a result our findings may not be generalisable to units with fewer health professionals or a less experienced team. The high burden of depression, and its modest responsiveness to treatment is increasingly recognized in the dialysis literature. We were therefore not surprised to find little correlation with physical outcomes and MCS scores, however we also acknowledge that our findings may in part relate to the small study sample, and to the nature of the KDQoL assessment of mental health wellbeing.

The data are limited by several factors. We did not include a validation cohort or long-term follow up data. A total of 12 patients did not have FIM data at baseline or follow up for unclear reasons. Another 37 did not have discharge FIM scores, mostly because these patients became acutely unwell and were transferred emergently to another acute care institution. Thus it is possible that our results are biased towards the inclusion only of those doing well with rehabilitation. Similarly a substantial number of patients did not complete both admission and discharge quality of life questionnaires limiting generalizability. Our study is also of relatively small size, and may not be generalizable across units with smaller teams or fewer health disciplines.

## Conclusion

We propose that the use of a simple staff subjective evaluation is a valid clinical tool that can be easily used to assess improvement in functional and physical aspects after rehabilitation. As little to no change was seen in the mental health scores on the KDQoL, it remains unclear whether the evaluation tool is able to capture changes in emotional well-being, were they to have occurred. Further studies are needed to determine whether this subjective evaluation can be extended to other patient subgroups.

## Data Availability

The datasets used and/or analysed during the current study available from the corresponding author on reasonable request.

## Appendix

**Table 2:** Examples of patient-rehabilitation Goals identified at admission

To be able to walk from sitting room to bathroom in current home setting with or without a cane (42 m).	
To be able to safely transfer in and out of the backseat of Ambassador taxi cab to wheelchair post-dialysis with either no assistance or one person supervision	
To safely manage erythropoietin administration in home setting	
To be able to transfer between dialysis chair and wheelchair with 1-person assist	
To be able to walk 300 m with/without walker	
To be able to safely manage outdoor step (home setting)	
To climb 6 stairs (after installation of new indoor stair rail)	
To maintain balance using two-feet stance for 4 min (to allow opening of condominium door lock)	
To be able to toilet independently	
To reduce fall risk in bathroom while performing daily grooming tasks	
To feed independently using nondominant hand (train in use of modified dishes and cutlery)	
To use non verbal communication skills eg picture charts	
To be able to get to church twice per week	
To control pain in L Knee	

## Abbreviations

FIM®: Functional Independence Measure
HD: Hemodialysis
IQR: Interquartile range
KDQoL: Kidney Disease Quality of Life score
MCS: Mental component score
QOL: Quality of life
PCS: Physical component score
